# Effect of Lactated Ringer Administration on Survival Outcomes in Critically Ill Patients With Acute Kidney Injury: A Retrospective Cohort Study

**DOI:** 10.1155/emmi/5576804

**Published:** 2025-04-08

**Authors:** Shengling Huang, Wenxue Liang, Yingxue Zhong, Shangjia Huang, Liangmei Chen, Donge Tang, Yunyi Li, Shuang Cui, Lingjun Shen, Bing Yan, Lianghong Yin, Fanna Liu

**Affiliations:** ^1^Department of Nephrology, The First Affiliated Hospital of Jinan University, Guangzhou 510627, China; ^2^Department of Gastrointestinal Surgery, First People's Hospital of Foshan, Foshan 528000, China; ^3^Department of Nephrology, Shenzhen First People's Hospital, Shenzhen 518000, China

**Keywords:** acute kidney injury, lactated Ringer's, restricted mean survival time, survival

## Abstract

**Background:** Although lactated Ringer's (LR) solution is widely used in managing patients with acute kidney injury (AKI), its specific impact on mortality remains unclear. This retrospective cohort study aimed to evaluate the effects of LR administration on survival outcomes in severely ill patients with AKI.

**Methods:** Critically ill patients with AKI were identified using data from the Medical Information Mart for Intensive Care-IV (MIMIC-IV) database. Propensity score matching (PSM) was employed to address baseline discrepancies between patients who received LR and those who did not. The association of LR administration with survival, duration of hospitalization and intensive care unit (ICU) stay, requirement for renal replacement therapy (RRT), renal function recovery, and hyperkalemia was analyzed using restricted mean survival time (RMST), logistic regression, and linear regression models.

**Results:** A total of 5620 patients with AKI were included. Following PSM, LR administration was associated with prolonged survival at 28 and 90 days compared to non-LR use (28-day survival increase: 1.12 days, 95% confidence interval [CI] 0.62–1.63, *p* < 0.001; 90-day survival increase: 3.73 days, 95% CI 1.70–5.76, *p* < 0.001). The survival benefit became more pronounced, with higher LR use linked to more remarkable 90-day survival. However, LR administration did not significantly affect renal function recovery or hyperkalemia incidence.

**Conclusion:** Administering LR to critically ill patients with AKI was associated with improved survival at both 28 and 90 days.

## 1. Introduction

The global prevalence of acute kidney injury (AKI) varies substantially, influenced by factors such as whether it is hospital-acquired (h-AKI) or community-acquired (c-AKI). AKI affects approximately 7%–18% of hospitalized patients [1] and occurs at a rate of 20–200 cases per million in the general population [2]. In ICUs, AKI substantially contributes to morbidity and mortality [3, 4], with a mean prevalence ranging from 30% to 50% [5]. The primary causes of AKI in patients hospitalized in the ICU are sepsis and hypovolemia [6, 7]. Timely fluid administration stabilizes hemodynamics in critically ill patients with anuric AKI by restoring intravascular volume, increasing stroke volume, and improving perfusion pressure. These effects improve renal perfusion and glomerular function [8]. However, uncertainties remain regarding the advantages of fluid therapy in individuals with AKI [9, 10]. Legrand et al. reported that active fluid resuscitation did not significantly impact renal microvascular blood flow or oxygen delivery in a hemorrhagic shock rat model [9]. Current evidence does not support fluid resuscitation as a means to improve AKI prognosis [11–13]. A multicenter observational cohort study suggested that a positive fluid balance might increase AKI-related mortality [12]. Moreover, fluid administration beyond the correction of hypovolemia can lead to adverse outcomes. For decades, 0.9% sodium chloride (saline) has been the most commonly used intravenous fluid. However, the chloride present in saline has been associated with renal vasoconstriction [14], inflammation [15], AKI [16], hyperchloremic metabolic acidosis [17], and eventually, increased mortality [18]. As a balanced crystalloid fluid, LR mimics the plasma's electrolyte composition and has recently gained popularity as a substitute for saline. Current studies among critically ill adults suggest that, compared to saline, balanced crystalloids (with LR being the primary formulation) are associated with lower mortality rates, reduced need for RRT, and a decreased incidence of renal dysfunction [19, 20]. However, the benefits of LR in improving ICU patient outcomes remain uncertain. Two large, single-center randomized controlled trials (RCTs)—the Saline versus Albumin for the Treatment of Patients with Decompensated Cirrhosis and Acute Kidney Injury: An Evaluation of Diuretic Response and Renal Outcome (SALT-ED) and Sepsis Management with Activated Recombinant Thrombomodulin (SMART) trials—investigated the use of balanced crystalloids, primarily LR, compared to saline in adults. Both trials reported no significant difference in mortality rates between the two treatment options [21, 22]. Previous research on balanced solutions has focused on specific patient groups, such as those with systemic inflammatory response syndrome (SIRS) [23], trauma [24], sepsis [20], or patients undergoing surgical procedures [25, 26]. However, studies specifically focusing on patients with AKI remain limited. Therefore, this study aimed to evaluate the impact of LR administration on mortality outcomes in patients experiencing AKI using data from the MIMIC-IV database.

## 2. Methodology

### 2.1. Data Source

This retrospective cohort study utilized data from the MIMIC-IV (version 1.0) database, which includes over 200,000 emergency ICU admissions at Beth Israel Deaconess Medical Center between 2008 and 2019 [27]. The lead author, Shengling Huang, completed the Collaborative Institutional Training Initiative Examination and obtained approval for database access (record ID: 42251770). The data extraction code is publicly available on GitHub (https://github.com/MIT-LCP/mimic-iv). Per the Privacy Rule of the Health Insurance Portability and Accountability Act, individual patient consent was waived since all data were anonymized to ensure privacy.

### 2.2. Participants

Patients were diagnosed with AKI based on the Kidney Disease: Improving Global Outcomes (KDIGO) criteria [28]. The baseline serum creatinine (SCr) level was defined as either the lowest SCr value recorded within 7 days before admission or, if unavailable, the initial SCr measurement obtained upon admission [29]. According to KDIGO clinical practice guidelines, AKI staging was determined by evaluating urine output and creatinine levels within the first 48 h of ICU admission [28].

Inclusion criteria included: (1) analysis of data from the initial ICU admission for individuals with multiple admissions; (2) age ≥ 18 years; and (3) clinical diagnosis of AKI within 48 h of ICU admission.

Exclusion criteria included: (1) hospital discharge within 48 h of ICU admission, (2) mortality within 48 h of ICU admission, (3) pre-existing diagnosis of chronic kidney disease stage 5 (CKD5), (4) receipt of RRT before screening, (5) history of renal transplantation, (6) administration of crystalloid fluids before AKI diagnosis, and (7) total crystalloid infusion volume of < 2 L within 24 h after AKI diagnosis.

### 2.3. Data Collection

Data for relevant variables on the first day of ICU admission were extracted from the MIMIC-IV database. These included demographic characteristics, sequential organ failure assessment (SOFA) score, simplified acute physiology score II (SAPS II), systolic and diastolic blood pressure, heart rate, mean arterial pressure (MAP), vasopressor use, respiratory rate, body temperature, body weight, oxygen saturation (SpO_2_), SCr, baseline creatinine, chloride, potassium, bicarbonate, anion gap, mechanical ventilation status, RRT use, and anchor year group.

AKI patients were categorized into two groups: the “LR” group, individuals administered with LR solution (with or without saline or other crystalloid solutions, and the “non-LR” group), and patients who were administered only non-LR crystalloids, including saline and other crystalloid solutions. The proportion of LR solution administered was calculated by dividing the daily volume of LR solution by the total daily volume of all crystalloids. Missing data points were excluded from the analysis (Additional file 1: Table S1).

### 2.4. Primary and Secondary Outcomes

This study primarily aimed to evaluate the 28-day survival rate, with secondary endpoints including the 90-day survival rate, length of stay (LOS) in the ICU and hospital, peak potassium concentration (hyperkalemia) during the ICU stay, recovery of renal function, and initiation of new RRT. The term “28-day survival” refers to patients who remained alive 28 days after ICU admission, while “90-day survival” denotes survival up to 90 days post-ICU admission. Recovery of kidney function was defined as a significant reduction in creatinine levels to less than 1.5 times the baseline values, along with normalized urinary output exceeding 0.5 mL/kg/h over a continuous 24-h period at discharge [30].

### 2.5. Restricted Mean Survival Time (RMST) Analysis

The RMST is a measure of the average survival time within a specified observation period (t), offering a meaningful alternative for survival analysis in studies with limited follow-up or right-censored data. By focusing on survival time up to a pre-determined timepoint, RMST circumvents the need to evaluate the entire survival curve and enhances the interpretability of survival outcomes. This method provides a reliable framework for analyzing survival outcomes in scenarios where complete long-term data are unavailable, thereby improving the interpretability of survival studies under practical constraints [31, 32]. RMST represents an absolute measure of survival time, offering a more intuitive alternative to hazard ratios (HRs) for clinicians and patients. RMST does not depend on the proportional hazards (PH) assumption, unlike Cox PH models. Concentrating on survival time within a predefined interval enables meaningful comparisons even when the proportionality of hazards is violated [33, 34]. RMST is calculated as the cumulative survival probability represented by the Kaplan–Meier (KM) curve over a specified time frame [35]. This approach reduced the impact of data from a small number of patients during later follow-up periods, thereby strengthening the robustness and reliability of the analysis.

### 2.6. Statistical Analysis

Categorical variables were presented as frequencies (percentages), and intergroup comparisons were conducted using appropriate statistical tests, such as the chi-square (χ^2^) test or Fisher's exact test. Continuous variables were expressed as either the mean with standard deviation or the median with its interquartile range, depending on the distribution. Group comparisons utilized the *t*-test or the Mann–Whitney *U* test as appropriate.

Propensity score matching (PSM) was performed at a 1:1 ratio to minimize baseline characteristic discrepancies between the LR and non-LR cohorts. Propensity scores were calculated using a logistic regression model, with a caliper width set to 0.2 standard deviations to optimize matching.

The association between LR use and extended survival time in patients with AKI was assessed using RMST analysis. The RMST values, their differences, ratios, and corresponding 95% CIs were calculated for 28- and 90-day intervals following ICU admission using the “survRM2” package in R software.

A logistic regression model examined the relationship between LR administration and outcomes associated with renal function recovery, hyperkalemia, and new RRT initiation. Results were reported as HRs alongside their respective 95% CIs. The effect of LR use on ICU and hospital LOS was assessed using a linear regression model, with HRs calculated applying the formula HR = *e*^βi^.

Subgroup analyses and sensitivity analysis were conducted to assess the robustness of results and explore potential dose–response relationships between LR administration rates and survival outcomes. The analyses were stratified by age, gender, daily fluid intake, AKI stage, sepsis-3 status, and the proportion of LR administration. All statistical analyses were conducted using the R package (version 4.2.1), with statistical significance defined as a *p*-value < 0.05.

## 3. Results

**Figure 1:**
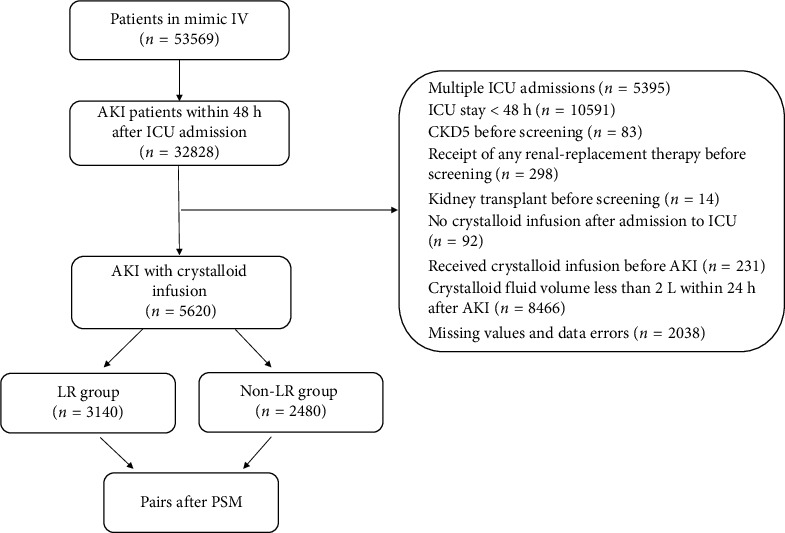
Flowchart of patient selection for the study. MIMIC-IV = medical information mart for intensive care-IV, AKI = acute kidney injury, ICU = intensive care unit, CKD5 = diagnosis of chronic kidney disease stage 5, PSM = propensity score matching.

The MIMIC-IV database identified 32,828 patients diagnosed with AKI. After applying exclusion criteria, a final cohort of 5620 individuals was chosen for this study. Of these, 3140 individuals received LR during their ICU stays, while 2480 did not (Figure 1). We conducted the Anderson–Darling (A–D) test for key continuous variables, and the results are shown in Table S4.

Table 1 summarizes the baseline characteristics of the LR and non-LR groups. Before PSM, considerable differences were observed between the two cohorts concerning gender, admission categories, anchor year group, and AKI stage. Compared to the non-LR group, the LR group exhibited the following characteristics: (1) higher heart rate, SCr, baseline creatinine, chloride, and potassium levels, along with elevated SAPS II and SOFA scores; (2) greater likelihood of receiving more significant volumes of pre-AKI fluids, total fluid input, colloid input, and mechanical ventilation (72.5% vs. 67.3%); (3) lower MAP, respiratory rate, fluid output, bicarbonate levels, and vasopressor use (40.5% vs. 56.2%); and (4) reduced incidence of heart failure (21.4% vs. 25.0%) and liver disease (11.8% vs. 14.4%). After PSM, the standardized mean difference (SMD) was less than 0.1, indicating a significant reduction in the baseline variables in both groups (Figure 1S).

### 3.1. Primary Outcomes

**Figure 2:**
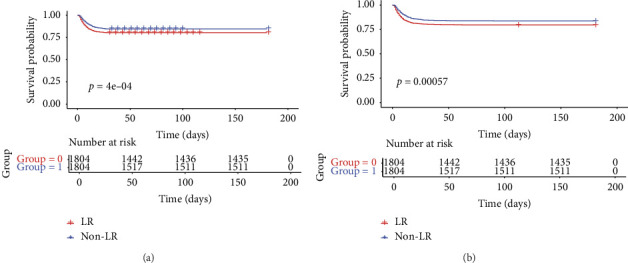
Kaplan–Meier survival curve after PSM. PSM = propensity score matching. The Kaplan–Meier estimates of survival time among 28 days (a) and 90 days (b) after PSM.

The RMST analysis was employed because it violated the PH assumption and the relatively low mortality rates in the LR and non-LR groups. In the pre-matched cohort, the expected survival times for the LR and non-LR groups during the initial 28 days before ICU admission were 25.25 days (95% CI 25.00–25.49) and 24.10 days (95% CI 23.76–24.42), respectively (Table 2). Over the 28-day period, the LR cohort had a mean survival advantage of 1.15 days (95% CI: 0.74–1.56) compared to the non-LR cohort. In the post-matched cohort, the LR group demonstrated a survival benefit of 1.12 days (95% CI 0.62–1.63; *p* < 0.001; Table 2). RMST analysis revealed a significant increase in 28-day survival time in the LR cohort. The KM curves for 28-day survival post-PSM are presented in Figure 2(a).

### 3.2. Secondary Outcomes

During the 90-day follow-up period, patients in the LR cohort had an RMST of 77.52 days (95% CI: 76.50–78.53), which was substantially longer than the 73.74 days (95% CI: 72.45–75.02) observed in the non-LR cohort (Table 2). This outcome indicated a significant survival improvement of 3.78 days (95% CI: 2.15–5.42). Following matching, the KM curve, shown in Figure 2(b), confirms that LR was associated with a 90-day survival advantage of 3.73 days (95% CI: 1.70–5.76). The RMST analysis demonstrated that the survival benefit of LR became more pronounced with longer follow-up durations.

Logistic regression analysis was used to assess the differences in renal function recovery, hyperkalemia, and new RRT outcomes between the two groups. Clinically considerable differences were observed in hyperkalemia and new RRT outcomes after PSM (Table 2). The impact of LR use on ICU and hospital LOS was assessed using a linear regression model. Before PSM (Table 2), the LOS in the LR group had a longer LOS compared to the non-LR group for both the ICU (5.0 vs. 4.6 days) and hospital (12.40 vs. 10.2 days). Following PSM, the LR group showed a more pronounced difference in ICU (4.8 vs. 4.6 days) and hospital (11.1 vs. 10.6 days) LOS.

### 3.3. Subgroup Analysis

**Figure 3:**
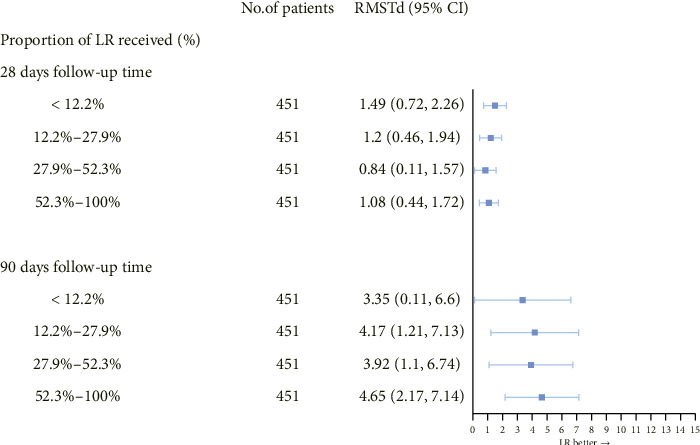
Dose–response relationship between balanced crystalloids and survival time.

Analysis based on the proportion of LR solution administered indicated a significant correlation between higher proportions of LR administration and improved 90-day survival. However, no significant association was observed between LR proportion and 28-day survival (Figure 3 and Additional file 1: Table S2). Subgroup analysis indicated a positive correlation between LR use and survival time, particularly in patients with AKI (Table 3). Patients aged 36–55 and older than 65 showed significantly longer survival. Furthermore, the RMST difference was more significant in men than women (1.47 vs. 0.80 days and 5.09 vs. 2.79 days, respectively).

Table 3 Subgroup analysis of LR use and clinical outcomes after PSM. RMST = restricted mean survival time. RMSTd = the difference of restricted mean survival time between the two groups (RMST_LR_ − RMST_Non-LR_).

Stratification by daily fluid input volume revealed improved outcomes in patients receiving medium to high daily fluid input volumes (> 1930 mL), with no significant benefit seen in those with low fluid volumes (≤ 1930 mL). The most substantial survival difference was observed in patients with a daily fluid intake of 2430–3020 mL (Table 3). Patients meeting the Sepsis-3 criteria exhibited significantly enhanced survival compared to those who did not. Subgroup analyses before PSM yielded consistent results (Additional file 1: Table S3).

### 3.4. Sensitivity Analysis

Sensitivity analysis included 10,591 patients who died within 48 h of ICU admission, 231 who received crystalloid infusion before AKI diagnosis, and 8466 patients who received < 2 L of crystalloid fluid within 24 h of AKI diagnosis. The results showed that LR administration was significantly associated with improved survival outcomes in patients with AKI (mean survival increase: 0.57 days, 95% CI 0.31–0.83, *p* < 0.001). In the sensitivity analysis, the Cox PH model demonstrated a similar trend (HR = 0.63, 95% CI: 0.56–0.72).

## 4. Discussion

This cohort study established a substantial correlation between LR administration and improved survival at both 28 days and 90 days in critically ill patients with AKI. Moreover, the benefits of LR became more evident with extended follow-up periods. Subgroup analysis revealed that patients receiving higher proportions of LR experienced better 90-day survival outcomes. Moreover, there was a substantial difference in new RRT incidence between the two cohorts, while no considerable differences were observed in renal function recovery and hyperkalemia. Furthermore, patients treated with LR had slightly longer hospital LOS and ICU than those receiving non-LR solutions.

The correlation between LR fluid resuscitation and survival in critically ill patients with AKI is novel. This study provides new evidence supporting LR as a potentially more effective fluid choice for these patients, aligning with the findings from a meta-analysis [36] and supporting data from a retrospective study [19]. A meta-analysis of 13 high-quality clinical RCTs found that the impact of balanced crystalloids on patient outcomes varied, showing a relative reduction in 90-day mortality by 9% and an increase of 1% compared to saline [36]. A retrospective cohort study indicated that higher LR use was associated with reduced mortality, influenced by the total volume of fluid volume administered [19]. The SMART trial, which involved 15,802 critically ill adults, showed a mortality rate of 10.3% in the balanced crystalloids versus 11.1% in the saline group (*p*=0.06) [37]. Similarly, the SALT trial, including 974 adults admitted to the ICU, reported no significant difference in in-hospital mortality between the two groups [38]. Conversely, our study demonstrated a significant survival difference. Several factors may explain this discrepancy: First, previous studies predominantly focused on critically ill and surgical patients in the ICU when investigating the association between crystalloid resuscitation fluids and survival outcomes [37–39]. Unlike the postoperative cohorts in the SPLIT trial [39] and patients with sepsis or respiratory failure in the SALT trial [38], our study targets explicitly severely ill patients with AKI. Second, the choice of balanced crystalloid solutions varied across studies. For example, the Balanced Solutions in Intensive Care Study (BASICS) [37] and 0.9% Saline vs Plasma-Lyte 148 (PL-148) for ICU Fluid Therapy (SPLIT) trials [39] compared balanced crystalloids, such as Plasma-Lyte 148. Third, our study's large, retrospective cohort drawn from the MIMIC-IV database may also account for the observed differences.

Saline administration has been linked to higher mortality rates and increased RRT requirements in critically ill adults compared to balanced crystalloids, as evidenced by previous studies [20, 40, 41]. Moreover, results from retrospective research made in the United States revealed that patients who received saline infusion on the day of abdominal surgery exhibited an elevated risk of mortality, acidosis, and need for RRT [42]. Moreover, a supporting analysis of the SMART trial found that balanced crystalloids were not associated with severe hyperkalemia but were significantly correlated with a reduced incidence of new RRT in individuals diagnosed with AKI [43]. This study corroborates previous findings, showing significant differences in outcomes related to new RRT. The current study proposed that the higher RRT rates in the saline group might be attributed to reduced renal blood flow caused by elevated chloride levels [44] and delayed urination associated with saline use [45]. However, chloride in both patient groups in this study contradicts these hypotheses. Treatment decisions regarding RRT initiation may have been influenced by clinician bias. Moreover, the small number of new RRT recipient cases likely introduced assessment bias due to an insufficient sample size.

Balanced crystalloids contain small amounts of potassium, which raises concerns about hyperkalemia, discouraging their use by clinicians. A recent retrospective observational clinical study found no independent association between balanced fluid use and hyperkalemia in patients with impaired kidney function [46]. Similarly, another cohort study revealed that balanced crystalloids were associated with lower incidences of severe hyperkalemia and acidosis compared to saline solutions [47]. The findings of these studies align with our analysis, indicating that LR may be safer than saline in managing potassium levels and minimizing electrolyte disturbances. Furthermore, our study found no evidence to suggest that LR use improved the likelihood of renal function recovery compared to non-LR solutions. Potential explanations for this observation include (1) the absence of a universally accepted definition of AKI recovery, leading to inconsistent criteria and possible underestimation of patients who regained renal function [48], and (2) inappropriate fluid administration, including fluids through medications and nutritional support, which may result in fluid overload, venous congestion, and direct renal parenchymal injury [49]. This finding is significant as it suggests that LR does not have a detrimental effect on kidney recovery in AKI patients, addressing concerns about the potential adverse effects of fluid therapy on renal function.

One of the key strengths of this study lies in its use of RMST analysis. In the sensitivity analysis, the Cox PH model demonstrated a similar trend. However, the PH assumption was violated (*p* < 0.05), further supporting our choice of RMST as the primary analytical method. This method has demonstrated utility in fields, such as cardiovascular medicine, diabetology [35], and oncology [34], offering a robust and interpretable approach to assessing treatment effects in clinical research where HR is unsuitable. Furthermore, the study provides critical insights into fluid management for critically ill patients with AKI, a population that has historically been underrepresented in fluid therapy research.

However, this study has several limitations. First, as a nonrandomized retrospective analysis, residual confounding could not be eliminated despite employing PSM. Second, clinical decisions regarding the timing of RRT initiation may have been subject to treatment bias. Third, the study could not determine the underlying causes or etiologies of AKI, potentially leaving confounding factors unaddressed. Fourth, the database lacked information on pre-admission fluid administration in the ICU, which may impact the validity of the findings. Lastly, sensitivity analyses revealed differences in secondary outcomes (hyperkalemia and the incidence of new RRT) compared to the primary analysis. This discrepancy may stem from including special populations, increasing patient heterogeneity and clinical complexity. Future clinical trials are necessary to explore the sources of fluid exposure and further validate these findings.

## 5. Conclusion

In summary, our study suggests that LR administration improves survival in ICU-admitted patients with AKI based on retrospective data. However, LR did not enhance renal function recovery. These findings hold significant clinical implications for choosing crystalloid infusion solutions for critically ill patients with AKI. Additional multicenter RCTs are required to validate these findings.

## Figures and Tables

**Table 1:** Baseline characteristics between two groups.

Variables	Before PSM				After PSM			
	Non-LR (*n* = 2480)	LR (*n* = 3140)	*p*	SMD	Non-LR (*n* = 1804)	LR (*n* = 1804)	*p*	SMD
Age, median (IQR), yr	66.0 (55.0, 77.0)	67.0 (55.0, 78.0)	0.450	0.008	66.0 (55.0, 77.0)	68.0 (55.0, 79.0)	0.484	0.012
Gender, male, *n* (%)	1437 (57.9)	1730 (55.1)	0.035	0.057	1024 (56.8)	1027 (56.9)	0.946	0.003
Ethnicity, white, *n* (%)	1593 (64.2)	2094 (66.7)	0.149	0.052	1179 (65.4)	1156 (64.1)	0.707	0.028
Admission type, emergency, *n* (%)	1570 (63.3)	1755 (55.9)	< 0.001	0.218	1084 (60.1)	1098 (60.9)	0.961	0.018
Weight, median (IQR), kg	83.8 (70.1, 99.5)	82.1 (69.1, 98.4)	0.395	0.003	83.0 (70.0, 99.1)	81.9 (68.5, 97.9)	0.352	0.003
Heart rate, median (IQR), bpm	84.4 (73.5, 97.3)	88.4 (77.2, 100.8)	< 0.001	0.204	86.1 (75.0, 98.5)	86.5 (75.4, 98.2)	0.910	0.007
MAP, median (IQR), mmHg	78.0 (71.6, 85.8)	75.6 (70.3, 82.6)	< 0.001	0.198	77.2 (71.1, 84.4)	76.5 (70.9, 84.3)	0.605	0.012
Respiratory rate, median (IQR), bpm	19.2 (16.9, 22.0)	18.9 (16.7, 21.8)	0.021	0.051	19.0 (16.7, 21.9)	19.0 (16.7, 21.8)	0.739	0.009
Temperature, median (IQR), °C	37.0 (36.7, 37.4)	37.0 (36.7, 37.3)	0.415	0.005	36.9 (36.7, 37.4)	37.0 (36.7, 37.3)	0.467	0.018
SpO_2_, median (IQR), %	97.5 (96.0, 98.8)	97.5 (96.1, 98.8)	0.546	0.010	97.4 (96.0, 98.8)	97.4 (96.0, 98.7)	0.968	0.005
Before AKI input, median (IQR), ml^a^	1770.0 (600.0, 3547.8)	2298.0 (812.8, 4577.0)	< 0.001	0.232	1959.5 (670.3, 3908.8)	1959.5 (700.0, 3814.8)	0.806	0.003
Before AKI colloid input, median (IQR), ml^b^	1213.5 (350.0, 2429.8.0)	1244.0 (393.8, 2541.3)	0.140	0.050	1299.5 (365.5, 2535.8)	1209.0 (407.0, 2450.5)	0.854	0.001
Bicarbonate, median (IQR), mmol/L	23.0 (21.0, 26.0)	22.0 (19.0, 25.0)	< 0.001	0.271	23.0 (20.0, 25.0)	23.0 (20.0, 25.0)	0.693	0.010
Creatinine, median (IQR), mg/dL	0.9 (0.7, 1.2)	1.0 (0.7, 1.3)	0.006	0.077	0.9 (0.7, 1.3)	0.9 (0.70, 1.3)	0.540	0.018
Baseline creatinine, median (IQR), mg/dL	0.9 (0.7, 1.2)	1.0 (0.7, 1.3)	0.017	0.067	0.9 (0.7, 1.2)	0.9 (0.70, 1.2)	0.496	0.027
Chloride, median (IQR), mg/dL	104.0 (101.0, 107.0)	105.0 (102.0, 109.0)	< 0.001	0.263	105.0 (101.0, 108.0)	104.0 (101.0, 108.0)	0.865	0.008
Aniongap, median (IQR), mg/dL	15.0 (12.0, 17.0)	14.0 (12.0, 17.0)	0.131	0.046	14.0 (12.0, 17.0)	14.0 (12.0, 17.0)	0.392	0.015
Potassium, median (IQR), mg/dL	4.0 (3.70, 4.5)	4.1 (3.70, 4.6)	< 0.001	0.133	4.1 (3.7, 4.5)	4.1 (3.7, 4.5)	0.648	0.012
SAPS II score	37.0 (29.0, 47.0)	40.0 (31.0, 49.3)	< 0.001	0.169	38.0 (30.0, 48.0)	38.0 (30.0, 48.0)	0.750	0.002
SOFA score	6.0 (4.0, 8.0)	6.0 (4.0, 9.0)	< 0.001	0.133	6.0 (4.0, 9.0)	6.0 (4.0, 9.0)	0.780	0.002
Fluid input, median (IQR), ml^c^	3401.0 (2746.0, 4305.0)	4221.0 (3277.0, 5644.8)	< 0.001	0.588	3629.0 (2941.5, 4572.0)	3611.0 (2989.5, 4594.3)	0.708	0.005
Colloid input, median (IQR), ml^d^	0.0 (0.0, 0.0)	0.0 (0.0, 350.0)	< 0.001	0.346	0.0 (0.0, 0.0)	0.0 (0.0, 0.0)	0.072	0.028
Fluid output, median (IQR), ml^e^	1289.5 (827.8, 1946.0)	1125.0 (740.0, 1675.5)	< 0.001	0.192	1223.5 (793.0, 1855.0)	1222.0 (776.8, 1770.0)	0.455	0.017
Anchor year group, *n* (%)			< 0.001	0.214			0.860	0.029
2008–2010	883 (35.6)	943 (30.0)			611 (33.9)	594 (32.9)		
2011–2013	629 (25.4)	726 (23.1)			422 (23.4)	432 (23.9)		
2014–2016	583 (23.5)	728 (23.2)			429 (23.8)	421 (23.3)		
2017–2019	385 (15.5)	743 (23.7)			342 (19.0)	357 (19.8)		
AKI stage, *n* (%)			0.014	0.078			0.358	0.048
1	1869 (75.4)	2271 (72.3)			1333 (73.9)	1358 (75.3)		
2	587 (23.7)	846 (26.9)			458 (25.4)	428 (23.7)		
3	24 (1.0)	23 (0.7)			13 (0.7)	18 (1.0)		
Diabetes, *n* (%)	699 (28.2)	839 (26.7)	0.233	0.033	494 (27.4)	486 (26.9)	0.793	0.010
Sepsis3, *n* (%)	1746 (70.4)	2378 (75.7)	< 0.001	0.120	1317 (73.0)	1311 (72.7)	0.852	0.007
Heart failure, *n* (%)	621 (25.0)	672 (21.4)	< 0.001	0.086	424 (23.5)	424 (23.5)	1.000	0.001
Renal disease, *n* (%)	333 (13.4)	437 (13.9)	0.623	0.014	234 (13.0)	244 (13.5)	0.659	0.016
Liver disease, *n* (%)	356 (14.4)	371 (11.8)	0.005	0.075	251 (13.9)	249 (13.8)	0.962	0.003
Chronic pulmonary disease, *n* (%)	596 (24.0)	781 (24.9)	0.486	0.020	425 (23.6)	445 (24.7)	0.460	0.026
Cancer, *n* (%)	325 (13.1)	473 (15.1)	0.040	0.056	255 (14.1)	261 (14.5)	0.812	0.010
Rheumatic disease, *n* (%)	76 (3.1)	120 (3.8)	0.143	0.042	63 (3.5)	64 (3.5)	1.000	0.003
Vasopressors use, *n* (%)	1394 (56.2)	1271 (40.5)	< 0.001	0.319	909 (50.4)	922 (51.1)	0.689	0.014
Mechanical ventilation								
Invasive, *n* (%)	1668 (67.3)	2276 (72.5)	< 0.001	0.114	1257 (69.7)	1254 (69.5)	0.942	0.004
Noninvasive, *n* (%)	85 (3.4)	148 (4.7)	0.020	0.065	69 (3.8)	62 (3.4)	0.593	0.021

Abbreviations: AKI = acute kidney injury, MAP = mean arterial pressure, RRT = renal replacement therapy, SAPS II = simplified acute physiology score II, SMD = standardized mean difference, SOFA = sequential organ failure assessment.

^a^Before AKI fluid input volume was recorded prior to diagnosis of AKI after ICU admission.

^b^Before AKI colloid input volume was recorded prior to diagnosis of AKI after ICU admission.

^c^Fluid input volume was recorded within the first 24 h after AKI diagnosis after ICU admission.

^d^Fluid output volume was recorded within the first 24 h after AKI diagnosis after ICU admission.

^e^Colloid input volume was recorded within the first 24 h after AKI diagnosis after ICU admission.

**Table 2:** Association of LR use with clinical outcomes in critically ill patients with AKI.

Before PSM	Non-LR *n* = 2480	LR *n* = 3140	RMSTd^b^/HR^c^	95% CI	*p*
RMST^a^ for 28 days (days)	24.10 (23.76, 24.42)	25.25 (25.00, 25.49)	1.15	0.74, 1.56	< 0.001
RMST for 90 days (days)	73.74 (72.45, 75.02)	77.52 (76.50, 78.53)	3.78	2.15, 5.42	< 0.001
LOS ICU (days)	4.6 (2.9, 8.2)	5.0 (3.1, 9.7)	0.81	0.46, 1.15	< 0.001
LOS hospital (days)	10.2 (6.5, 16.6)	12.4 (7.76, 19.9)	1.89	1.21, 2.56	< 0.001
Recovery of renal function (%)	1901 (44.7%)	2354 (55.3%)	0.91	0.81, 1.03	0.144
Hyperkalemia (%)	316 (36.5%)	550 (63.5%)	1.02	1.01, 1.04	0.009
New RRT (%)	93 (41.0%)	134 (59.0%)	1.14	0.87, 1.50	0.328
After PSM (1:1)	1804	1804			
RMST for 28 days (days)	24.04 (23.65, 24.43)	25.16 (24.84, 25.49)	1.12	0.62, 1.63	< 0.001
RMST for 90 days (days)	73.54 (72.03, 75.06)	77.28 (75.93, 78.63)	3.73	1.70, 5.76	< 0.001
LOS ICU (days)	4.6 (3.0, 8.6)	4.8 (3.0, 8.6)	0.72	0.32, 1.11	< 0.001
LOS hospital (days)	10.6 (6.7, 17.1)	11.1 (7.3, 17.7)	1.40	0.63, 2.17	< 0.001
Recovery of renal function (%)	1345 (49.6%)	1368 (50.4%)	1.07	0.92, 1.25	0.375
Hyperkalemia (%)	242 (46.4%)	279 (53.6%)	1.18	0.98, 1.40	0.080
New RRT (%)	85 (61.6%)	53 (38.4%)	0.56	0.37, 0.86	0.008

Abbreviations: AKI = acute kidney injury, HR = hazard ratio, ICU = intensive care unit, LOS = lengths of stay, PSM = propensity score matching, RMST = restricted mean survival time, RRT = renal replacement therapy.

^a^RMST represents the restricted mean survival time for each group within the first 28 and 90 days after admission to the ICU.

^b^RMSTd means the difference of restricted mean survival time between the two groups (RMST_LR_-RMST_Non-LR_).

^c^HR was calculated using the formula HR = e^βi^.

**Table 3:** Subgroup analysis of the association between LR use and clinical outcome after PSM.

Subgroup	*N*	Difference in RMST^a^ among 28 days		Difference in RMST among 90 days	
		RMSTd^b^ (95% CI)	*p*	RMSTd (95% CI)	*p*
Age (yr)					
18–35	224	0.15 (−1.05, 1.35)	0.803	0.67 (−4.24, 5.58)	0.789
36–55	706	1.33 (0.48, 2.17)	0.002	5.66 (2.29, 9.03)	0.001
56–65	736	0.73 (−0.21, 1.67)	0.129	1.17 (−2.54, 4.87)	0.539
> 65	1942	1.44 (0.72, 2.16)	< 0.001	4.94 (2.11, 7.78)	0.001
Gender (%)					
Female	1557	0.80 (0.10, 1.51)	0.026	2.79 (0.00, 5.57)	0.050
Male	2051	1.47 (0.90, 2.05)	< 0.001	5.09 (2.79, 7.39)	< 0.001
AKI stage (%)					
1	2691	0.97 (0.44, 1.4)	< 0.001	3.25 (1.17, 5.32)	< 0.001
≥ 2	917	1.94 (1.06, 2.82)	< 0.001	6.80 (3.26, 10.35)	< 0.001
Sepsis3 (%)					
Yes	2628	1.59 (1.08, 2.10)	< 0.001	5.49 (3.46, 7.53)	< 0.001
No	980	1.01 (0.25, 1.76)	0.009	3.80 (1.01, 6.59)	0.008
Daily fluid input (ml)^c^					
(632, 1930]	902	0.48 (−0.44, 1.40)	0.306	1.47 (−2.29, 4.58)	0.512
(1930, 2430]	902	1.14 (0.28, 2.01)	0.009	4.26 (0.71, 7.81)	0.019
(2430, 3020]	902	1.97 (1.08, 2.85)	< 0.001	7.32 (3.82, 10.83)	< 0.001
(3020, 6840]	902	1.51 (0.55, 2.48)	0.002	5.01 (1.22, 8.80)	0.010

Abbreviations: AKI = acute kidney injury, PSM = propensity score matching, RMST = restricted mean survival time.

^a^RMST represents the restricted mean survival time for each group within the first 28 and 90 days after admission to the ICU.

^b^RMSTd means the difference of restricted mean survival time between the two groups (RMST_LR_-RMST_Non-LR_).

^c^Daily fluid input was recorded as every 24 h after AKI diagnosis during the ICU stay.

## Data Availability

The MIMIC IV database (version 1.0) is publicly available from https://mimic-iv.mit.edu/. The codes used in the manuscript are available from https://github.com/MIT-LCP/mimic-IV.

## Nomenclature

AKI: Acute kidney injury
MIMIC-IV: Medical information mart for intensive care-IV
PSM: Propensity score matching
RRT: Renal replacement therapy
ICU: Intensive care unit
RCT: Randomized controlled trial
KDIGO: Kidney disease: improving global outcomes
SCr: Serum creatinine
CKD5: Chronic kidney disease stage 5
SOFA: Sequential organ failure assessment
SAPS II: Simplified acute physiology score II
MAP: Mean arterial pressure
LOS: Length of stay
RMST: Restricted mean survival time
SMDs: Standardized mean differences
PH: Proportional hazards
A-D: Anderson–Darling

## Supporting Information

Supporting Information 1 Table S1: Missing number (%) for variables.

Supporting Information 2 Figure 1S: Distribution of propensity scores in two groups.

Supporting Information 3 Table S2: Dose–response relationship between LR administration and survival time before PSM.

Supporting Information 4 Table S3: Subgroup analysis before PSM.

Supporting Information 5 Table S4: Normality test.
