# Supplementary material for: Effect of Lactated Ringer Administration on Survival Outcomes in Critically Ill Patients With Acute Kidney Injury: A Retrospective Cohort Study
Source: Emerg Med Int. 2025 Apr 8;2025:5576804. doi: 10.1155/emmi/5576804 (PMC11999744; doi:10.1155/emmi/5576804)
Supplement: Supporting Information 1 — Table S1: Missing number (%) for variables. [file 5576804.f1.docx]

**Table S1 Missing number (%) for variables**

| **Risk variables** | **Missing number (%)** |
| --- | --- |
| Temperature | 387(77.4) |
| Weight | 22(4.4) |
| Aniongap | 10(2.0) |
| Chloride | 10(2.0) |
| Bicarbonate | 10(2.0) |
| Potassium | 10(2.0) |
| Creatinine | 9(1.8) |
| Baseline Creatinine | 9(1.8) |
| Respiratory rate | 5(1.0) |
| Spo2 | 4(0.8) |
| MAP | 3(0.6) |
| Heart rate | 3(0.6) |
| Age | 0 |
| Gender | 0 |
| Race | 0 |
| Admission type | 0 |
| SAPS II score | 0 |
| SOFA | 0 |
| Fluid input | 0 |
| Colloids input | 0 |
| Fluid output | 0 |
| Before AKI input | 0 |
| Before AKI colloids input | 0 |
| Anchor year group | 0 |
| AKI stage | 0 |
| Diabetes | 0 |
| Sepsis3 | 0 |
| Heart failure | 0 |
| Renal disease | 0 |
| Liver disease | 0 |
| Chronic pulmonary disease | 0 |
| Cancer | 0 |
| Rheumatic disease | 0 |
| Vasopressors use | 0 |
| Invasive | 0 |
| Noninvasive | 0 |
| **Outcome variables** |  |
| Los ICU | 0 |
| Los hospital | 0 |
| Admittime | 0 |
| RRT | 0 |
| Death time | 0 |
| Kidney function recover | 0 |
| Max potassium | 18(3.6) |

MAP=mean arterial pressure, SAPS II= simplified acute physiology score II, SOFA=sequential organ failure assessment, LOS=lengths of stay, ICU=intensive care unit, RRT= renal replacement therapy, AKI= acute kidney injury.
