# Supplementary figures and images for: Effect of Lactated Ringer Administration on Survival Outcomes in Critically Ill Patients With Acute Kidney Injury: A Retrospective Cohort Study
Source: Emerg Med Int. 2025 Apr 8;2025:5576804. doi: 10.1155/emmi/5576804 (PMC11999744; doi:10.1155/emmi/5576804)

Figure 1S: Distribution of propensity scores in two groups

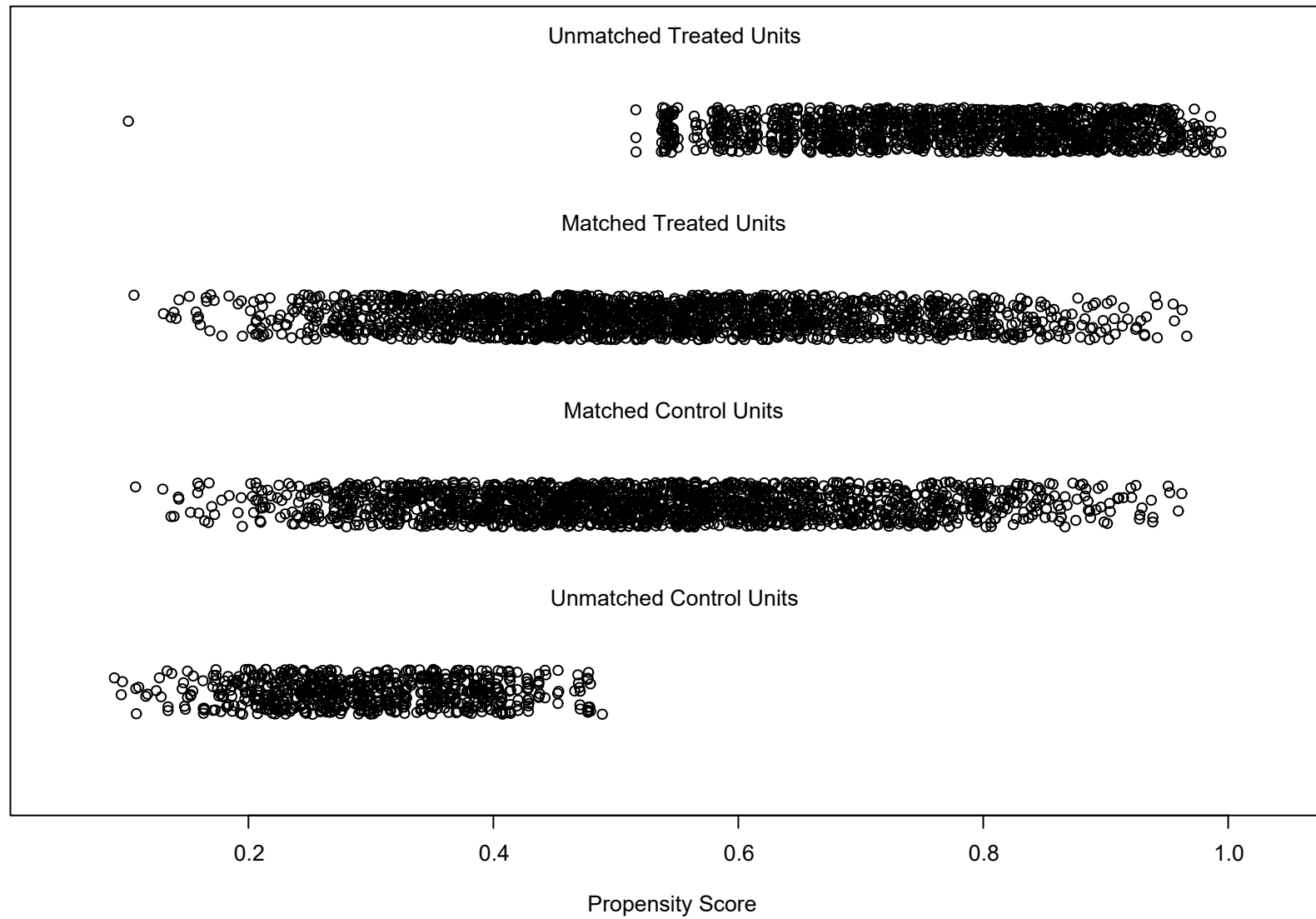

Supplement: Supporting Information 2 — Figure 1S: Distribution of propensity scores in two groups. [file 5576804.f2.pdf]
