# Supplementary material for: Effect of Lactated Ringer Administration on Survival Outcomes in Critically Ill Patients With Acute Kidney Injury: A Retrospective Cohort Study
Source: Emerg Med Int. 2025 Apr 8;2025:5576804. doi: 10.1155/emmi/5576804 (PMC11999744; doi:10.1155/emmi/5576804)
Supplement: Supporting Information 3 — Table S2: Dose–response relationship between LR administration and survival time before PSM. [file 5576804.f3.docx]

**Table S2** **dose-response relationship between LR administration and survival time before PSM.**

| Proportion of LR received(%) | N | Difference in RMST^a^ among 28-day | | Difference in RMST among 90-day | |
| --- | --- | --- | --- | --- | --- |
|  |  | RMSTd^b^(95%CI) | *P* | RMSTd(95%CI) | *P* |
| 0 | 2480 | 0 |  | 0 |  |
| ＜13.4% | 785 | 1.84(1.24,2.43) | <0.001 | 5.27(2.81,7.74) | <0.001 |
| 13.4%-30.2% | 785 | 1.52(0.96,2.09) | <0.001 | 5.45(3.19,7.71) | <0.001 |
| 30.7%-53.7% | 785 | 1.35(0.78,1.93) | <0.001 | 5.59(3.35,7.84) | <0.001 |
| 53.7%-100% | 785 | 2.56(2.06,3.06) | <0.001 | 10.39(8.52,12.25) | <0.001 |

RMST= restricted mean survival time.

^a^RMST represents the restricted mean survival time for each group within the first 28 and 90 days after admission to the ICU.

^b^RMSTd means the difference of restricted mean survival time between the two groups (RMST_LR_- RMST_Non-LR_).
