# Supplementary material for: Effect of Lactated Ringer Administration on Survival Outcomes in Critically Ill Patients With Acute Kidney Injury: A Retrospective Cohort Study
Source: Emerg Med Int. 2025 Apr 8;2025:5576804. doi: 10.1155/emmi/5576804 (PMC11999744; doi:10.1155/emmi/5576804)
Supplement: Supporting Information 4 — Table S3: Subgroup analysis before PSM. [file 5576804.f4.docx]

**Table S3 Subgroup analysis before PSM.**

| Subgroup | N | Difference in RMST^a^ among 28-day | | Difference in RMST among 90-day | |
| --- | --- | --- | --- | --- | --- |
|  |  | RMSTd^b^(95%CI) | *P* | RMSTd(95%CI) | *P* |
| Age(yr) |  |  |  |  |  |
| 18-35 | 353 | 0.88(0.25,2.01) | 0.127 | 3.53(0.92,7.98) | 0.120 |
| 36-55 | 1100 | 0.89(0.17,1.61) | 0.015 | 4.03(1.18,6.89) | 0.006 |
| 56-65 | 1147 | 0.94(0.14,1.74) | 0.021 | 2.49(0.64,5.61) | 0.119 |
| ＞65 | 3020 | 1.87(1.26,2.48) | <0.001 | 6.59(4.18, 9.00) | <0.001 |
| Gender(%) |  |  |  |  |  |
| Femal | 2453 | 1.44(0.83,2.04) | <0.001 | 5.32(2.96,7.67) | <0.001 |
| Mal | 3167 | 1.92(1.43,2.41) | <0.001 | 6.94(5.00,8.89) | <0.001 |
| AKI stage(%) |  |  |  |  |  |
| 1 | 4140 | 1.58(1.14,2.03) | <0.001 | 5.87(4.13,7.61) | <0.001 |
| >=2 | 1480 | 2.10(1.33,2.86) | <0.001 | 7.29(4.28,10.29) | <0.001 |
| Sepsis3(%) |  |  |  |  |  |
| Yes | 4124 | 1.78(1.321,2.25) | <0.001 | 6.47(4.61,8.34) | <0.001 |
| No | 1496 | 1.387(0.76,2.01) | <0.001 | 5.17(2.86,7.48) | <0.001 |
| Daily fluid input(ml)^a^ |  |  |  |  |  |
| (632,1960] | 1406 | 1.10(0.37,1.83) | 0.003 | 3.75(0.90,6.60) | 0.010 |
| (1960,2480] | 1404 | 0.94(0.24,1.65) | 0.008 | 3.86(1.00,6.71) | 0.008 |
| (1960,3090] | 1405 | 2.09(1.32,2.86) | <0.001 | 7.69(4.64,10.73) | <0.001 |
| (3090,6910] | 1405 | 2.63(1.74,3.51) | <0.001 | 9.25(5.84,12.66) | <0.001 |

RMST= restricted mean survival time, AKI= acute kidney injury, PSM=propensity score matching.

^a^RMST represents the restricted mean survival time for each group within the first 28 and 90 days after admission to the ICU.

^b^RMSTd means the difference of restricted mean survival time between the two groups (RMST_LR_- RMST_Non-LR_).

^a^Daily fluid input was recorded as every 24 hours after AKI diagnosis during the ICU stay.
