# Supplementary material for: Effect of Lactated Ringer Administration on Survival Outcomes in Critically Ill Patients With Acute Kidney Injury: A Retrospective Cohort Study
Source: Emerg Med Int. 2025 Apr 8;2025:5576804. doi: 10.1155/emmi/5576804 (PMC11999744; doi:10.1155/emmi/5576804)
Supplement: Supporting Information 5 — Table S4: Normality test. [file 5576804.f5.docx]

**Table S4 Normality Test**

| Variable | Test Method | Test Statistic | *P* | Normality Assumption |
| --- | --- | --- | --- | --- |
| Age | A-D test | 37.89 | ＜0.001 | Rejected (Non-normal) |
| Weight | A-D test | 47.35 | ＜0.001 | Rejected (Non-normal) |
| Heart rate | A-D test | 12.43 | ＜0.001 | Rejected (Non-normal) |
| Respiratory rate | A-D test | 45.53 | ＜0.001 | Rejected (Non-normal) |
| Temperature | A-D test | 19.77 | ＜0.001 | Rejected (Non-normal) |
| SpO2 | A-D test | 49.40 | ＜0.001 | Rejected (Non-normal) |
| Before AKI input | A-D test | 199.1 | ＜0.001 | Rejected (Non-normal) |
| Before AKI colloid input | A-D test | 212.79 | ＜0.001 | Rejected (Non-normal) |
| Bicarbonate | A-D test | 21.34 | ＜0.001 | Rejected (Non-normal) |
| Creatinine | A-D test | 187.36 | ＜0.001 | Rejected (Non-normal) |
| Baseline creatinine | A-D test | 193.3 | ＜0.001 | Rejected (Non-normal) |
| Chloride | A-D test | 17.99 | ＜0.001 | Rejected (Non-normal) |
| Aniongap | A-D test | 41.97 | ＜0.001 | Rejected (Non-normal) |
| Potassium | A-D test | 33.91 | ＜0.001 | Rejected (Non-normal) |
| SAPS II score | A-D test | 25.84 | ＜0.001 | Rejected (Non-normal) |
| SOFA score | A-D test | 75.03 | ＜0.001 | Rejected (Non-normal) |
| Fluid input | A-D test | 157.46 | ＜0.001 | Rejected (Non-normal) |
| Colloid input, | A-D test | 1023.9 | ＜0.001 | Rejected (Non-normal) |
| Fluid output | A-D test | 129.78 | ＜0.001 | Rejected (Non-normal) |

SAPS II= simplified acute physiology score II, SOFA=sequential organ failure assessment, AKI= acute kidney injury, A-D test =Anderson-Darling test.

A-D test was used for normality testing of the data distribution; *p* < 0.01 indicates non-normality.
